# A Method for Pedestrian Trajectory Prediction Using INS-GNSS Wearable Devices

**DOI:** 10.3390/s26041309

**Published:** 2026-02-18

**Authors:** Shengli Pang, Zhe Wang, Shiji Xu, Weichen Long, Ruoyu Pan, Honggang Wang

**Affiliations:** College of Communication and Information Engineering, Xi’an University of Posts and Telecommunications, Xi’an 710121, China; pangshengli@xupt.edu.cn (S.P.); xushiji@stu.xupt.edu.cn (S.X.); longweichen@stu.xupt.edu.cn (W.L.); panruoyu@xupt.edu.cn (R.P.); wanghonggang@xupt.edu.cn (H.W.)

**Keywords:** pedestrian trajectory prediction, INS-GNSS integration, wearable sensors, sensor fusion, adaptive Kalman filter

## Abstract

Driven by advancements in artificial intelligence technology, pedestrian trajectory prediction is shifting from traditional machine learning methods toward autonomous decision-making frameworks based on neural networks. However, the spatiotemporal uncertainty of pedestrian movement results in low accuracy of existing prediction models. To address this issue, we propose a multi-source perception fusion system based on INS-GNSS wearable devices. By integrating high-precision inertial measurement units (IMUs) and multi-mode global navigation satellite systems (GNSS), we enhance localization and prediction accuracy. For localization, we introduce a Gait Adaptive UKF (Gait-AUKF) that identifies pedestrian gait patterns and motion states by fusing multi-sensor data. An adaptive algorithm effectively suppresses trajectory drift and improves tracking accuracy. For trajectory prediction, we propose a pedestrian trajectory prediction framework based on a multi-source fusion attention mechanism. A GRU encoder extracts pedestrian trajectory features from historical motion data. An attention mechanism assigns varying weights to trajectory features across different scales. An LSTM decoder and A* path planning algorithm constrain spatiotemporal paths to generate future pedestrian trajectories. Experimental results demonstrate that compared to UKF and AKF, the Gait-AUKF reduces eastward error by 30%, northward error by 26.27%, and vertical error by 49.08%. The complete prediction framework achieves a 68.54% reduction in average position error (APE) and a 70.42% reduction in direction error (DE) compared to LSTM and Transformer models. Ablation experiments demonstrate that the integrated Gait-AUKF algorithm and A* path planning algorithm enhance model decision performance. After incorporating these algorithms, the model’s ADE decreased by 68.49% and FDE by 71.86%.

## 1. Introduction

In the fields of urban navigation and personal safety, reliable and precise pedestrian localization and tracking systems play an extremely critical role. Pedestrian trajectory prediction (PTP) [1] serves as a key technical prerequisite for ensuring the safety of vulnerable road users (VRUs) [2] in autonomous driving systems. Its core task is to model a pedestrian’s past and current movement data to infer their historical motion states, thereby predicting their future spatiotemporal path [3]. However, pedestrian motion is inherently highly random and multimodal due to intentional uncertainty [4], making accurate and robust trajectory prediction a persistent challenge, particularly in complex urban environments.

In pedestrian navigation, high-precision inertial measurement units (IMUs) can provide high-frequency motion data, but errors in IMU sensors accumulate over time [5,6,7]. Multi-global navigation satellite systems (Multi-GNSS) [8] can improve positioning accuracy, but signals are prone to loss in complex environments such as urban high-rise buildings and tunnels [9]. Therefore, the INS/GNSS integrated navigation system maintains accuracy in the event of signal loss through complementary advantages, while eliminating IMU accumulated errors through satellite positioning. The key lies in effectively integrating the two types of information [10,11]. To suppress IMU drift, IMUs are often placed on the feet or legs and zero velocity update (ZUPT) technology is used. Traditional ZUPT relies on a fixed threshold and is only suitable for uniform motion, making it difficult to adapt to different gaits. Therefore, in previous studies, Johan et al. [12] uased Bayesian adaptive thresholding method to select a separate threshold for each type of motion pattern, but this method overly relied on the number of motion patterns. Cho et al. [13] developed a threshold-free algorithm that detects zero speed through signal shape but is limited to walking and brisk walking motion patterns. This article proposes an adaptive unscented Kalman filter localization algorithm based on gait constraint model. By establishing a pedestrian gait phase constraint model and detecting zero velocity intervals, the heading and step size are optimized according to different motion states. Meanwhile, utilizing an adaptive Kalman filter to fuse motion data and correct position drift during gait effectively improves positioning accuracy and robustness in complex environments.

With the rapid development of intelligent transportation, urban planning, and public safety, trajectory prediction technology has gradually become one of the research hotspots. Pedestrian trajectory prediction is widely used in scenarios such as autonomous driving, crowd behavior analysis, and intelligent monitoring [14]. Due to human sociality, uncertainty in movement, and environmental factors, pedestrian trajectory prediction is a challenging task. In pedestrian trajectory prediction methods, there are mainly data-driven methods and motion model establishment. Firstly, most prediction methods rely on observable external stimuli [15], including historical trajectories, kinematic attributes (such as position, velocity, angular velocity), and contextual information such as road geometry and pedestrian vehicle interaction [16]. Secondly, modeling methods include parameterized methods based on kinematics and dynamics, as well as shallow and deep learning techniques [17,18]. These methods are optimized through various loss functions to generate outputs such as Gaussian distributions, multimodal trajectories, or probabilistic grids.

In recent years, the development of deep learning has significantly improved the accuracy and robustness of pedestrian trajectory prediction, making it increasingly important in practical applications such as autonomous driving and robot navigation. However, accurately modeling of the spatiotemporal relationships in pedestrian motion-especially when facing complex scenes and multimodal future behavior-remains a challenge. Early works such as the Social LSTM model proposed by Alahi et al. [19] integrated the hidden states of neighboring pedestrians through a grid based pooling mechanism, achieving preliminary modeling of pedestrian interaction. Gupta et al. [20] introduced generative adversarial networks (GANs) to handle the multimodal characteristics of trajectory prediction. However, these methods often rely on predefined interaction features or fixed neighborhood structures, making it difficult to explain the complex relationships between pedestrian trajectories. Li et al. [21] used an adaptive spatiotemporal graph construction algorithm to calculate edge weights based on dynamic features such as velocity and direction and combined them with temporal characteristics to generate more accurate trajectory predictions. The SHENet framework proposed by Meng et al. [22] uses a memory bank to store historical trajectories and establishes trajectory prediction based on the relationship between individuals and their surrounding environment. Li, R. et al. [23] combined multi-scale graph based spatial transformers and trajectory smoothing algorithms to predict multiple paths of historical trajectories. Although these advances have been made, there are still two issues with pedestrian trajectory prediction at present. Firstly, methods based on spatiotemporal graphs overly rely on interaction graphs with a single scale, ignoring the long-term trajectory relationships of pedestrians. Secondly, generative models perform well in terms of diversity but often lack clear mechanisms to ensure temporal consistency, resulting in high-frequency turns in generated paths and often ignoring pedestrian dynamic behavior.

Pedestrian trajectory prediction relies on current and historical trajectory information, detecting pedestrian motion intentions and states through motion behavior to enhance the accuracy of future trajectory prediction. This article proposes a multi-source data fusion behavioral attention mechanism framework for pedestrian trajectory prediction and path planning. Using INS and GNSS data to locate pedestrian trajectories, while capturing pedestrian gait motion information as guidance data for motion features. The framework uses a gated recurrent unit (GRU) encoder to extract key features of pedestrian motion and construct an adaptive fusion mechanism guided by physical constraints. Introducing a memory module to store pedestrian historical trajectories and assigning different attention weights to trajectory features at different scales through an attention mechanism addressable device. The LSTM decoder combines a spatiotemporal constraint path planning coordination mechanism to decode future pedestrian motion trajectories, achieving accurate prediction of pedestrian travel paths. The main contributions of this article are summarized as follows:

1. Existing gait assistance algorithms typically use ZUPT or PDR algorithms to estimate the step size and direction of two-dimensional plane displacement localization. The gait constraint localization method proposed in this article can distinguish gait changes and use ZUPT during the standing phase, which can not only update speed but also dynamically adjust the noise statistical characteristics of the filter. At the same time, a motion model for the swing phase was constructed to constrain and expand trajectory positioning in three-dimensional space. The method provides more detailed pedestrian gait decomposition and action model analysis, improving the accuracy of pedestrian trajectory localization.

2. Unlike visual or radar based prediction methods, the limitations of static images make it difficult to understand the spatiotemporal connections between pedestrian movements. By using wearable solutions, the complex spatial relationships of pedestrian movements can be easily captured for predicting low-cost pedestrian positions.

3. The method integrates pedestrian gait information into a unified framework for target tracking and future trajectory prediction, achieving end-to-end sharing of information and effectively addressing noise issues in practical scenarios.

4. The historical memory module is introduced into the attention mechanism, which ensures the smoothness and temporal consistency of predicted trajectories based on retrieval features and trajectory embedding.

The structure of this article is as follows: Section 2 introduces the design framework of the wearable devices used in the study. Section 3 introduces the gait constrained trajectory localization algorithm and pedestrian trajectory prediction algorithm framework proposed in this study. Section 4 introduces the results of algorithm simulation environment and experimental scenario testing. Section 5 is the research conclusion.

## 2. Wearable Device Design

**Figure 1:**
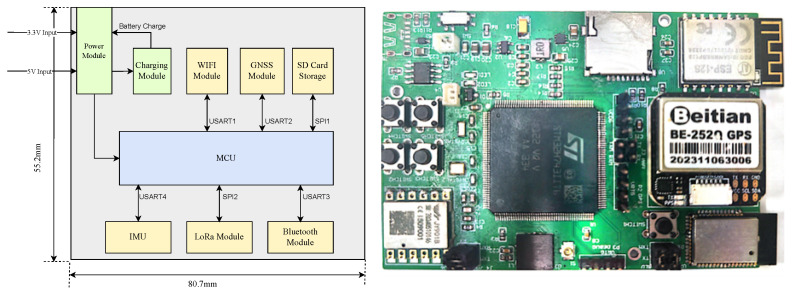
Wearable hardware design and physical diagram.

To accurately capture the dynamic behavior of pedestrians, this study designed a wearable data acquisition hardware device based on the INS-GNSS multi-mode integrated navigation system. The system is powered by a centralized rechargeable 3.7 V lithium battery. The hardware platform adopts STM32H7 series microcontrollers based on Cortex-M7 core, responsible for processing and parsing data from various sensors. The selected IMU is the JY-901S nine-axis inertial sensor, which integrates high-precision three-axis accelerometers, three-axis gyroscopes, and three-axis magnetometers and can collect pedestrian gait data at high frequencies. The selected GNSS module is E108-GN04D, which supports multi-system joint localization (BDS/GPS/GLONASS/Galileo) to provide accurate pedestrian position information. This device integrates multiple communication protocol modules, allowing for flexible information transmission based on specific scenarios. In addition, the collected pedestrian posture data and GNSS data can be stored on the SD memory card in the terminal device for subsequent analysis. The hardware architecture and physical diagram are shown in Figure 1, and the key parameters of IMU and GNSS are shown in Table 1 and Table 2.

## 3. Methodology

### 3.1. System Overview

**Figure 2:**
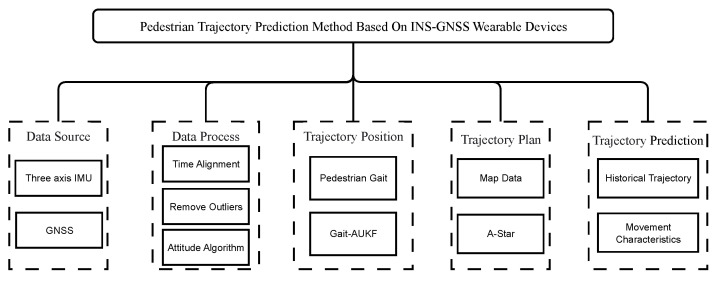
Overview of pedestrian trajectory prediction method.

The framework proposed in this article is an integrated framework that includes data perception, pedestrian gait analysis and localization, and pedestrian trajectory prediction. Its core is the principle of multi-source sensor fusion: fusing high-frequency IMU data with low-frequency but high-precision GNSS data to achieve stable and accurate pedestrian positioning and motion prediction. The main modules of the system are shown in Figure 2.

The system collects data obtained from wearable devices, filters it, and inputs it into the Gait-AUKF algorithm for high-precision 3D positioning by integrating IMU and GNSS data. Detailed gait analysis is also performed to obtain pedestrian gait action data, provide the data output by the algorithm to the prediction module, capture the dynamic characteristics of pedestrian motion through gate controlled loop units, and establish spatiotemporal correlations by compressing historical trajectories with memory enhanced attention mechanisms to guide future predictions. Based on a long short-term memory network, a future trajectory with reasonable behavior is generated, and the A* path planning algorithm is added to physically constrain the motion trajectory. The final predicted trajectory generated is both in line with the intention and feasible.

### 3.2. Pedestrian Dead Reckoning

Pedestrian dead reckoning (PDR) [24] is a sensor-based localization technique that estimates a pedestrian’s trajectory in real-time by detecting steps, estimating stride length, and determining heading angle. In this paper, wavelet transform is employed to distinguish gait phases, and the derived step frequency and stride length are incorporated into the PDR algorithm to enhance the accuracy of trajectory estimation. Given an initial position $\left(X_{0},Y_{0}\right)$, subsequent positions are computed recursively using the step length $L_{k}$ and the heading angle $\Psi_{k}$:

(1) $$\left\{\begin{array}{c} X_{k}=X_{k-1}+L_{k}\cos\Psi_{k}\\ Y_{k}=Y_{k-1}+L_{k}\sin\Psi_{k} \end{array}\right.$$

where $X_{k}$ and $Y_{k}$ denote the current position coordinates, *k* represents the step index, and k≥1. During walking, the magnitude of acceleration exhibits periodic fluctuations. For heading estimation, angular velocity $\omega ={\left[\omega_{x},\omega_{y},\omega_{z}\right]}^{T}$ acquired from a triaxial gyroscope is integrated numerically to obtain a short-term heading estimate. Observations from a triaxial magnetometer $m={\left[m_{x},m_{y},m_{z}\right]}^{T}$ are projected onto the horizontal plane using pitch and roll angles, thereby compensating for the accumulated integration error of the gyroscope through magnetometer-based correction. Equation (3) converts the magnetometer from the device coordinate system to the horizontal navigation coordinate system, where $H_{x}$ represents the X-direction magnetic field component on the horizontal plane, and $H_{y}$ represents the Y-direction magnetic field component on the horizontal plane. An adaptive weighting factor $\alpha_{w}\in \left[0,1\right]$ is introduced to balance the contributions of the two sensors:

(2) $$\Psi_{k}=\Psi_{k-1}+\int_{t_{k-1}}^{t_{k}}\omega_{z}\left(t\right)dt$$

(3) $$\left\{\begin{array}{c} H_{x}=m_{x}\cos\phi +m_{y}\sin\theta \sin\phi +m_{z}\cos\theta \sin\phi \\ H_{y}=m_{y}\cos\theta -m_{z}\sin\theta \end{array}\right.$$

(4) $$\Psi_{\mathrm{mag}}=\arctan\left(\frac{H_{y}}{H_{x}}\right)$$

(5) $$\Psi_{k}=\left(1-\alpha_{w}\right)\cdot \Psi_{k}+\alpha_{w}\cdot \Psi_{\mathrm{mag}}$$

### 3.3. Adaptive Unscented Kalman Filter Algorithm

The Kalman Filter is an optimal recursive estimation algorithm based on Bayesian estimation theory, suitable for linear Gaussian systems. Its core concept lies in a predictionupdate cycle that integrates a system dynamics model with noisy measurement data to achieve minimum mean square error estimation of the state variables. The state and observation equations are given by Equations (6) and (7), respectively:

(6) $$x_{k}=F_{k|k-1}x_{k-1}+B_{k}u_{k}+w_{k}$$

(7) $$z_{k}=H_{k}x_{k}+v_{k}$$

(8) $$\left\{\begin{array}{c} E\left[w_{k}\right]=0,\mathrm{cov}\left(w_{k},w_{j}\right)=Q_{k}\delta_{kj}\\ E\left[v_{k}\right]=0,\mathrm{cov}\left(v_{k},v_{j}\right)=R_{k}\delta_{kj}\\ \mathrm{cov}\left(w_{k},v_{j}\right)=0 \end{array}\right.$$

where $x_{k},z_{k}$ denote the state vector and observation vector at time *k*, $F_{k|k-1}$ is the state transition matrix, and $H_{k}$ is the observation matrix. The process noise $w_{k}∼N\left(0,Q_{k}\right)$ and the measurement noise $v_{k}∼N\left(0,R_{k}\right)$, where $Q_{k}$ and $R_{k}$ represent the process and measurement noise covariance matrices, respectively. Their statistical properties are specified as Formula (8).

The unscented Kalman filter addresses the nonlinear propagation of mean and covariance through the unscented transform (UT), which employs a deterministic sampling strategy [25]. A set of sigma points is generated and propagated through the nonlinear function, after which the mean and covariance of the transformed points are computed to approximate the output statistics. For a discrete-time nonlinear system, the UKF is formulated as follows:

(9) $$\left\{\begin{array}{c} X_{k}=f\left(X_{k-1},u_{k},W_{k}\right)\\ Z_{K}=H_{k}X_{k}+V_{k} \end{array}\right.$$

where $f\left(X\right)$ is a nonlinear state transition function, $H_{k}$ is the observation function, and $W_{k}$,$V_{k}$ are uncorrelated zero-mean Gaussian white noise processes. Here, $q_{k}$ is a non-negative definite matrix and $r_{k}$ is a positive definite matrix, representing the covariance matrices of $W_{k}$ and $V_{k}$, respectively. The Kronecker delta function is denoted by $\delta_{kj}$.

(10) $$\left\{\begin{array}{c} E\left[W_{k}\right]=0,\mathrm{cov}\left(W_{k},W_{j}\right)=q_{k}\delta_{kj}\\ E\left[V_{k}\right]=0,\mathrm{cov}\left(V_{k},V_{j}\right)=r_{k}\delta_{kj}\\ \mathrm{cov}\left(W_{k},V_{j}\right)=0 \end{array}\right.$$

Algorithms estimate the state of nonlinear dynamic systems through a series of structured processes. The process begins with the initialization of state estimation and covariance matrix:

(11) $${\hat{x}}_{0}=E\left[x_{0}\right],P_{0}=E\left[\left(x_{0}-{\hat{x}}_{0}\right){\left(x_{0}-{\hat{x}}_{0}\right)}^{T}\right]$$

where ${\hat{x}}_{0}$ is the initial state vector and $P_{0}$ is a positive symmetric definite covariance matrix. Subsequently, a set of 2n+1 sigma points is generated, where denotes the dimension of the state vector. Given the state mean ${\hat{x}}_{k-1}$ and covariance $P_{k-1}$ at time k−1, the sigma points are computed as

(12) $$\left\{\begin{array}{c} X_{k-1}^{\left(0\right)}={\hat{x}}_{k-1}\\ X_{k-1}^{\left(i\right)}=X_{k-1}^{\left(0\right)}+\gamma {\left(\sqrt{P_{k-1}}\right)}_{i}i=1,2,\ldots ,n\\ X_{k-1}^{\left(i\right)}=X_{k-1}^{\left(0\right)}-\gamma {\left(\sqrt{P_{k-1}}\right)}_{i}i=n+1,n+2,\ldots ,2n \end{array}\right.$$

Here, $\gamma =\sqrt{n+\lambda }$, $\lambda =\alpha^{2}\left(n+\zeta \right)-n$. α, ζ is a scaling parameter that controls the spread of the sigma points around the mean. During the time update, each sigma point is propagated through the nonlinear process model:

(13) $$X_{k|k-1}^{*}=f\left(X_{k-1},u_{k}\right)$$

The predicted state mean ${\hat{x}}_{k}^{-}$ and covariance $P_{k}^{-}$ are then computed as

(14) $${\hat{x}}_{k}^{-}=\sum_{i=0}^{2n}W_{i}^{\left(m\right)}X_{i,k|k-1}^{*}$$

(15) $$P_{k}^{-}=\sum_{i=0}^{2n}W_{i}^{\left(c\right)}\left(X_{i,k|k-1}^{*}-{\hat{x}}_{k}^{-}\right){\left(X_{i,k|k-1}^{*}-{\hat{x}}_{k}^{-}\right)}^{T}+Q_{k-1}$$

where *Q* is the process noise covariance matrix, and $W_{i}^{\left(m\right)}$ and $W_{i}^{\left(c\right)}$ are weights assigned to the mean and covariance calculations, respectively.

(16) $$\begin{array}{c} W_{0}^{\left(m\right)}=\frac{\lambda }{n+\lambda } \end{array}$$

(17) $$\begin{array}{c} W_{0}^{\left(c\right)}=\frac{\lambda }{n+\lambda }+\left(1-\alpha^{2}+\beta \right) \end{array}$$

(18) $$\begin{array}{c} W_{i}^{\left(m\right)}=W_{i}^{\left(c\right)}=\frac{1}{2(n+\lambda )},\;i=1,\ldots ,2n \end{array}$$

For the measurement update, the observation sigma points are generated and transformed using the measurement model:

(19) $$Z_{k|k-1}=h\left(X_{k|k-1}^{*}\right)$$

The predicted measurement mean ${\hat{z}}_{k}^{-}$, innovation covariance $P_{zz,k}$, and cross-covariance $P_{xz,k}$ are calculated as follows:

(20) $${\hat{z}}_{k}^{-}=\sum_{i=0}^{2n}W_{i}^{\left(m\right)}Z_{i,k|k-1}$$

(21) $$\nu_{k}=z_{k}-{\hat{z}}_{k}^{-}$$

(22) $$P_{zz,k}=\sum_{i=0}^{2n}W_{i}^{\left(c\right)}\left(Z_{i,k|k-1}-{\hat{z}}_{k}^{-}\right){\left(Z_{i,k|k-1}-{\hat{z}}_{k}^{-}\right)}^{T}+R_{k-1}$$

(23) $$P_{xz,k}=\sum_{i=0}^{2n}W_{i}^{\left(c\right)}\left(X_{i,k|k-1}^{*}-{\hat{x}}_{k}^{-}\right){\left(Z_{i,k|k-1}-{\hat{z}}_{k}^{-}\right)}^{T}$$

Finally, the Kalman gain $K_{k}$ is computed, and the state estimate and covariance are updated:

(24) $$K_{k}=P_{xz,k}P_{zz,k}^{-1}$$

(25) $${\hat{x}}_{k}={\hat{x}}_{k}^{-}+K_{k}\nu_{k}$$

(26) $$P_{k}=P_{k}^{-}-K_{k}P_{zz,k}K_{k}^{T}$$

This formulation ensures an efficient and accurate mechanism for state estimation in nonlinear systems through sigma point propagation and statistical linearization.

The adaptive unscented Kalman filter (AUKF) [26] incorporates a mechanism for adaptive tuning of the noise covariance matrices. The algorithm proposed in this paper further integrates an adaptive adjustment strategy based on the statistical characteristics of the innovation sequence, enabling dynamic optimization of filtering parameters in response to real time changes in pedestrian motion. The adaptive factor is computed using the norm of the normalized innovation sequence and implemented via a piecewise function for gradual adjustment. The normalized innovation sequence is defined as

(27) $$v_{k}=z_{k}-{\hat{z}}_{k|k-1},{\bar{v}}_{k}=P_{zz}^{-1/2}v_{k}$$

where $v_{k}$ is the original innovation sequence and ${\bar{v}}_{k}$ is its normalized form. A piecewise adaptive factor is designed based on the norm of the normalized innovation sequence:

(28) $$\alpha_{k}=\left\{\begin{array}{cc} 1, & \mathrm{if}\;||{\bar{v}}_{k}\left|\right|\leq c_{0}\\ \frac{c_{0}}{\left|\right|{\bar{v}}_{k}\left|\right|}\cdot \frac{c_{1}-\left|\right|{\bar{v}}_{k}\left|\right|}{c_{1}-c_{0}}, & \mathrm{if}\;c_{0}<||{\bar{v}}_{k}\left|\right|\leq c_{1}\\ 0, & \mathrm{if}\;||{\bar{v}}_{k}\left|\right|>c_{1} \end{array}\right.$$

This function facilitates progressive adjustment of the filtering gain: when the innovation sequence exhibits normal statistical characteristics, standard filtering performance is maintained; when minor model mismatch is detected, the weight of measurement information is reduced proportionally; and in the presence of severe anomalies, the filter relies entirely on predicted information, effectively mitigating the impact of abnormal observations on localization accuracy. The process noise covariance is updated adaptively as:

(29) $${\hat{Q}}_{k}=\frac{1}{N}\sum_{j=k-N+1}^{k}K_{j}v_{j}v_{j}^{T}K_{j}^{T}$$

Similarly, the measurement noise covariance is adjusted via

(30) $${\hat{R}}_{k}=\frac{1}{N}\sum_{j=k-N+1}^{k}\left(v_{j}v_{j}^{T}-P_{zz,j}\right)$$

### 3.4. The Proposed Gait-AUKF Algorithm for Localization

**Figure 3:**
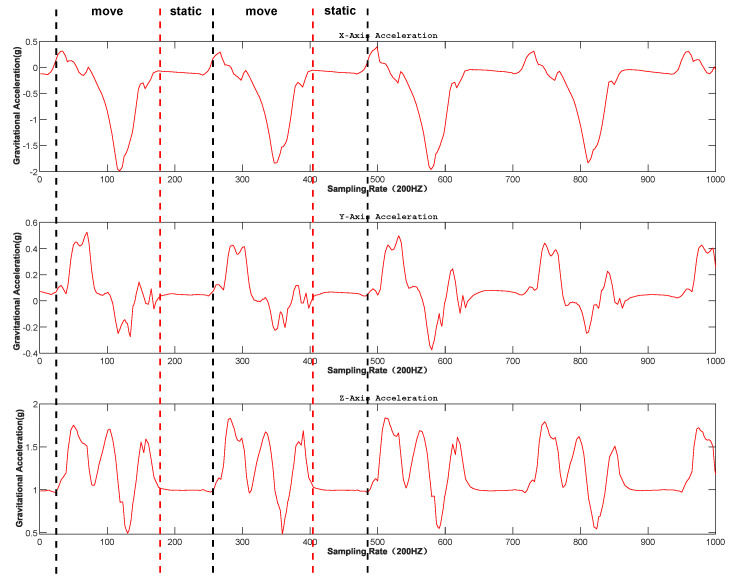
Pedestrian gait differentiation diagram.

This paper proposes a gait phase-constrained adaptive unscented Kalman filter (UKF) [27] localization algorithm that achieves high precision pedestrian localization by fusing INS data, GNSS measurements, and human gait characteristics [28]. First, a 16-dimensional state space model is established, encompassing position, velocity, attitude, and sensor biases. Second, a gait phase detection method based on acceleration signals is designed to accurately identify the stance and swing phases. Then, an adaptive UKF framework is constructed, where process and measurement noise covariances are dynamically adjusted by monitoring innovation sequences. Finally, a phase dependent constraint weight adjustment mechanism is introduced to apply velocity update constraints during different gait phases. Experimental results demonstrate that the proposed algorithm effectively suppresses drift errors from IMU sensors and maintains high localization accuracy even during GNSS signal outages. Figure 3 depicts the three-axis acceleration changes that transition between dynamic and static during gait.

The algorithm employs a 16-dimensional state vector $x={\left[p^{T},v^{T},q^{T},b_{a}^{T},b_{g}^{T}\right]}^{T}$ to represent the pedestrian’s motion state, including the position vector $p={\left[p_{x},p_{y},p_{z}\right]}^{T}$, velocity vector $v={\left[v_{x},v_{y},v_{z}\right]}^{T}$, attitude quaternion $q={\left[q_{w},q_{x},q_{y},q_{z}\right]}^{T}$, accelerometer bias $b_{a}={\left[b_{ax},b_{ay},b_{az}\right]}^{T}$, and gyroscope bias $b_{g}={\left[b_{gx},b_{gy},b_{gz}\right]}^{T}$.

The measurement inputs include INS data, GNSS position data, and acceleration based gait phase detection. The GNSS observation model measurement equation is as follows:

(31) $$z_{G}=\left[\begin{array}{c} P_{\mathrm{GNSS}}^{n}\\ v_{\mathrm{GNSS}}^{n} \end{array}\right]=H_{G}x_{k}+R_{G}$$

Gait discrimination is divided into two types: standing phase and swinging phase. The zero velocity update (ZUPT) mechanism is activated when the standing phase is detected, constraining the current velocity state to a zero vector and effectively reducing velocity drift. The gait phase function is defined as

(32) $$\phi \left(t\right)=\arctan2\left(\int_{0}^{t}∥a\left(\tau \right)∥\sin\left(\omega_{s}\tau \right)d\tau ,\int_{0}^{t}∥a\left(\tau \right)∥\cos\left(\omega_{s}\tau \right)d\tau \right)$$

ϕ(t) is the gait phase at time *t*, a(τ) is the acceleration at time τ, and $\omega_{s}$ is the gait angular frequency. When the standing phase is detected, the zero velocity constraint is applied:

(33) $$0=H_{Z}x_{k}+R_{Z}$$

(34) $$\left[\begin{array}{c} \alpha_{g}\cdot {\hat{v}}_{x}^{b}\\ \beta \cdot {\hat{v}}_{x}^{b}+\gamma_{g} \end{array}\right]=S\cdot C_{n}^{b}\left(q\right)\;v^{n}+R_{\mathrm{Gait}}$$

During the swing phase, use gait periodicity to establish a motion model related to forward speed or gait phase. The model is shown in the following formula. ${\hat{v}}_{x}^{b}$ indicate the estimated velocity in the x-axis direction in the carrier coordinate system, S used to select or combine specific components from three-dimensional velocity vectors, $\alpha_{g}$, $\gamma_{g}$ and β is gait related model parameters. $C_{n}^{b}\left(q\right)$ is the rotation matrix from the navigation coordinate system to the carrier coordinate system. After incorporating the subsequent process into the PDR and AUKF algorithms for multi-source observation fusion, it is fed back to the motion mechanics to form a closed loop for pedestrian position localization.

In contrast to the conventional UKF and even the standard AUKF, the proposed Gait-AUKF algorithm introduces a gait phase constraint mechanism that integrates PDR to estimate pedestrian trajectories. This allows the system to maintain localization accuracy even during GNSS outages. By incorporating gait phase detection, the algorithm provides additional reference information for trajectory prediction. It adaptively adjusts the noise statistical model to accommodate the diversity of pedestrian motion patterns, effectively handling uncertainties in complex scenarios such as gait transitions and turning maneuvers, thereby significantly enhancing localization accuracy in challenging environments.

### 3.5. A Multi-Source Attention Framework for Pedestrian Trajectory Prediction and Planning

#### 3.5.1. Framework Overview

This study proposes a hybrid framework for pedestrian trajectory prediction in complex dynamic environments, which integrates multi-source fusion attention mechanism, long short-term memory (LSTM) network, and A* path planning algorithm. This model is based on the front-end Gait-AUKF algorithm for real time estimation of pedestrian trajectory and gait information. By delving into the intrinsic correlation between leg movement features and advanced behavioral intentions, pedestrian trajectory prediction is achieved.

**Figure 4:**
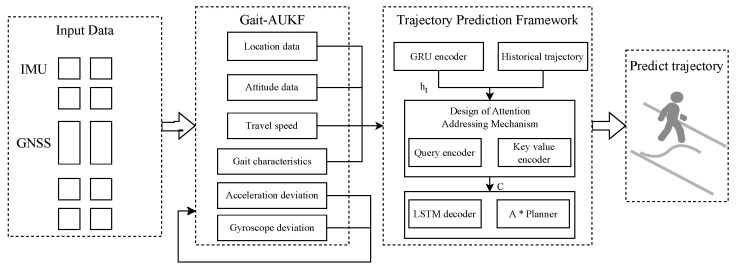
A multi-source attention framework for pedestrian trajectory prediction and planning.

In terms of architecture design, this model fully utilizes the advantages of attention mechanism in capturing long-distance spatiotemporal dependencies, as well as the strengths of long short-term memory (LSTM) network in maintaining motion state memory. By analyzing the temporal evolution characteristics such as gait cycle and step size, the system can simulate the probability distribution of pedestrian intention. On this basis, an A* path planning algorithm for collaborative reasoning is introduced: the A* path planning algorithm guides the pedestrian direction provided by the front-end, performs heuristic search in the environmental map, and generates the optimal or suboptimal geometric path that conforms to physical constraints such as obstacles and road structures. The environmental map is a map data from the experimental area, obtained by vectorizing and annotating information such as road boundaries, pedestrian crossings, and fixed obstacles. The output of the prediction module will undergo dynamic collaborative evaluation with the planned path, ultimately generating a trajectory that combines behavioral authenticity and physical feasibility. The pedestrian trajectory prediction architecture and data flow diagram are shown in Figure 4.

According to architecture Figure 4, it can be seen that the data flow of the algorithm begins with multi-source perception data from wearable devices, which is received and processed by the Gait-AUKF module to generate a 16-dimensional state vector and gait feature vector, including three-dimensional position, three-dimensional velocity, quaternion pose, and sensor deviation estimation. Sensor error estimation is used for module adaptive parameter adjustment. The original 16-dimensional state undergoes dimension and format conversion as a time series input to the GRU encoder, where acceleration is calculated from velocity difference. The GRU encoder compresses the temporal feature sequence into a fixed length encoding vector $h_{T}$ as the query state and sends it to the attention mechanism module. It interacts with the key value encoder in the historical memory to output a weighted context c. The LSTM decoder obtains the data stream and outputs the final predicted trajectory under the physical constraints of the A* path planner.

#### 3.5.2. Gated Recurrent Unit Encoding Mechanism

**Figure 5:**
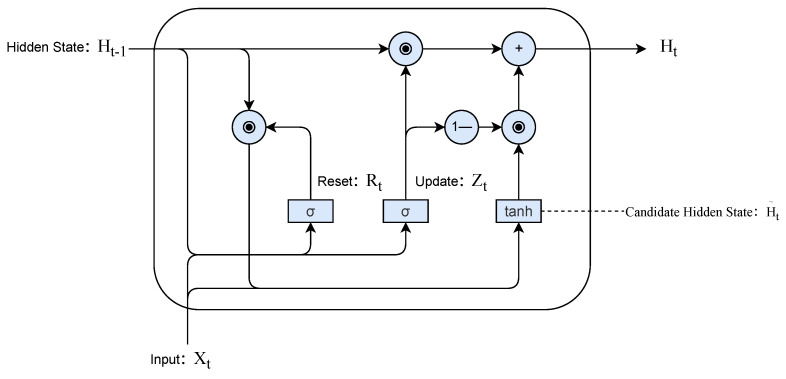
Structure of the gated recurrent unit (GRU).

The motion feature encoder is the temporal feature extraction module of this prediction model, which takes the real-time multi-dimensional pedestrian state sequence output by Gait-AUKF as input. Its core is a cyclic encoding network composed of gated recurrent units (GRUs). This encoder utilizes the gating mechanism of GRU to adaptively integrate the short-term dynamics and long-term trends of pedestrian trajectories, thereby constructing a hierarchical motion feature representation. The input data is the feature vector $x_{t}=\left[\;p_{t},\;v_{t},\;a_{t},\;g_{t}\;\right]$ for each time step, and GRU controls the flow of information through reset and update gates, as shown in Figure 5 where $p_{t}$ denotes the 2D planar coordinates; $v_{t}$ and $a_{t}$ represent the instantaneous velocity and acceleration vectors, respectively; and $g_{t}$ corresponds to the gait feature vector.

Resetting the gate can reduce the influence of historical states when gait mutations are detected, making candidate states more dependent on the current input and responding to instantaneous changes. When the motion trend of the update gate is stable, it tends to retain most of the historical state, thereby maintaining the continuity of the motion direction and supporting long-term path modeling. The encoder outputs the hidden state of the last time step as the representation of the entire sequence. This vector $h_{T}$ integrates multi-level information from subtle gait adjustments to macroscopic motion trends, which will be used as input for subsequent multi-source fusion attention modules for further spatiotemporal correlation and future trajectory inference.

#### 3.5.3. Design of the Attention-Based Addressing Mechanism

The memory enhanced attention addressing module is the core module for implementing historical experience retrieval in this framework. Its function is to establish semantic similarity mapping between the currently observed features and compressed historical memory features, thereby providing interpretable historical references for prediction. This module receives two input sources: first, the feature vector $q_{m}=h_{T}$ output by the motion feature encoder that fuses the current time period’s motion and intent, and second, the key value set $M={\left\{(k_{i},v_{i})\right\}}_{i=1}^{N}$ constructed by compressing and encoding the features of all historical time steps. The key vector $k_{i}$ is used for similarity addressing, and the value vector $v_{i}$ stores the corresponding trajectory context information.

This module adopts a dual encoder architecture to map the target pedestrian’s historical trajectory set and pedestrian action features to the same semantic space and combines the spatiotemporal relationship between pedestrian action patterns and historical trajectories to predict the pedestrian’s future trajectory. The query encoder takes pedestrian motion feature vectors as input and establishes a nonlinear mapping based on pedestrian motion features. The key value encoder takes pedestrian trajectory data as input, learns the periodic motion patterns of pedestrians in the trajectory, and captures long-term temporal dependencies. Query encoder and key value encoder are multi-layer perceptrons with parameter sharing, where $\theta_{q}$, $\theta_{k}$ and $\theta_{v}$ are learnable parameters, $s(q_{m},k_{i})$ represents the similarity score. In the calculation, since both $q^{\prime }$ and $k_{i}^{\prime }$ are normalized, Formula (35) can be optimized as a dot product.

(35) $$q^{\prime }=f_{q}\left(q_{m};\theta_{q}\right),\;k_{i}^{\prime }=f_{k}\left(k_{i};\theta_{k}\right)\;v_{i}^{\prime }=f_{v}\left(v_{i};\theta_{v}\right)$$

(36) $$s\left(q_{m},k_{i}\right)=\frac{q^{\prime }\cdot k_{i}^{\prime }}{∥q^{\prime }∥\;∥k_{i}^{\prime }∥}=q^{\prime }\cdot k_{i}^{\prime }$$

Normalize the similarity score to attention weights to achieve soft addressing of memory, $\alpha_{i}$ represents the attention weight of the *i* memory.

(37) $$\begin{array}{cc} \alpha_{i} & =\frac{\exp\left(s\left(q_{m},k_{i}\right)/\sqrt{d_{h}}\right)}{\sum_{j=1}^{N}\exp\left(s\left(q_{m},k_{j}\right)/\sqrt{d_{h}}\right)}. \end{array}$$

The context vector c retrieved is a weighted aggregation of memory values.

(38) $$\begin{array}{cc} c & =\sum_{i=1}^{N}\alpha_{i}\cdot v_{i}. \end{array}$$

The obtained vector c integrates the historical experience most relevant to the current state. This vector will be concatenated with the output of the GRU encoder to form an enhanced fusion feature.

#### 3.5.4. Collaboration Between LSTM Decoder Trajectory Prediction and A* Path Planning

**Figure 6:**
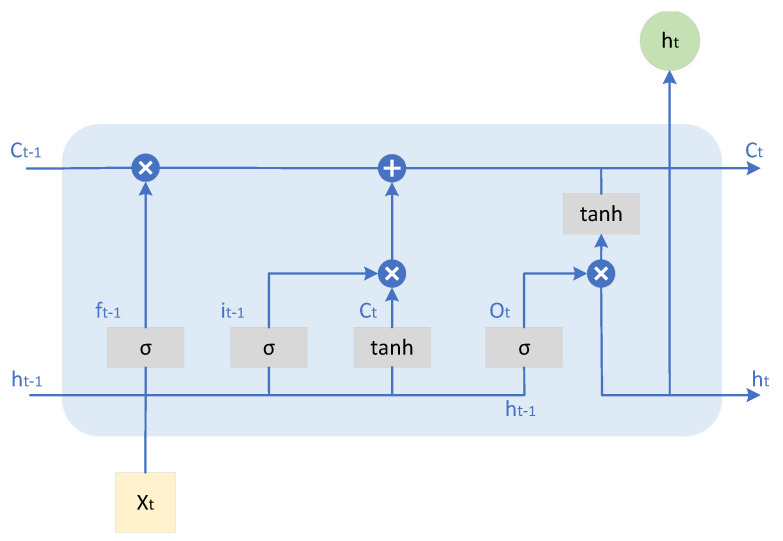
Schematic diagram of the LSTM structure.

This framework adopts a collaborative mechanism of LSTM decoder and A* path planning [29] to generate trajectory predictions that conform to pedestrian behavior patterns while ensuring physical feasibility and intention orientation. The LSTM decoder [30] takes the feature representation obtained in the encoding stage as the initial state input $h_{t}=\left[h_{T},c\right]$, and gradually generates the future trajectory sequence through recursion. The schematic diagram of recursive calculation is shown in Figure 6.

The A* path planning algorithm provides physically feasible path guidance for the LSTM decoder through heuristic search. The algorithm takes the current pedestrian position $P_{t}$ as the starting point, determines the target point $d_{t}=\left(\cos\theta_{t},\;\sin\theta_{t}\right)$ based on orientation information $P_{g}=P_{t}+L\cdot d_{t}$, and searches for the optimal path to avoid obstacles in the environment grid map. The core evaluation function balances the actual cost with heuristic estimation: f(n)=g(n)+h(n). Where g(n) is the actual cumulative cost from the starting point to node *n*, and h(n) is the Euclidean distance heuristic function. The algorithm outputs a sequence of path points $P^{\mathrm{astar}}$ as the intention guidance for pedestrians to move towards the target implicit in the path. The trajectory prediction $P^{\mathrm{lstm}}$ generated by the LSTM decoder is fused with the physically feasible path $P^{\mathrm{astar}}$ planned by the A* path planning algorithm through a collaborative weighting mechanism as follows.

(39) $$P_{t+k}=\sigma \left(\theta_{k}\right)\cdot P_{t+k}^{lstm}+\left(1-\sigma \left(\theta_{k}\right)\right)\cdot P_{t+k}^{\mathrm{astar}},k=1,\ldots ,K\;\theta_{k}=w_{1}\cdot u_{k}+w_{2}\cdot e^{-d_{k}}+b$$

The final trajectory coordinate $P_{t+k}$ is obtained by adaptively fusing the coordinates predicted by the LSTM decoder, $P_{t+k}^{\mathrm{lstm}}$, and the coordinates planned by the A* path planning algorithm, $P_{t+k}^{\mathrm{astar}}$, through weight fusion. The weights are calculated by the sigmoid function $\sigma (\theta_{k})$, $u_{k}$ is the variance based on prediction, and $d_{k}$ is the difference between prediction and planning.

## 4. Results and Discussion

### 4.1. Gait-AUKF Pedestrian Trajectory Localization Algorithm

**Figure 7:**
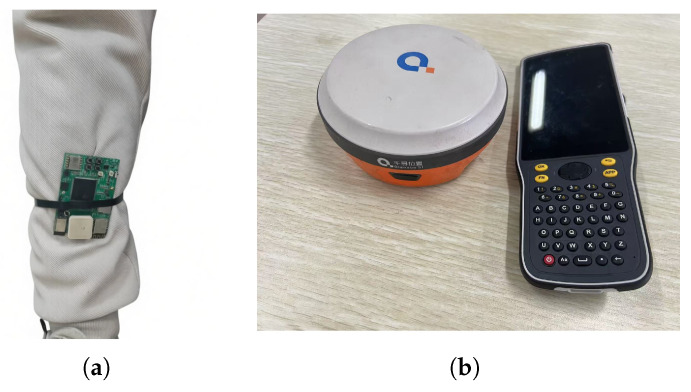
Wearable device placement and RTK equipment. (**a**) Placement of wearable device. (**b**) RTK equipment diagram.

**Figure 8:**
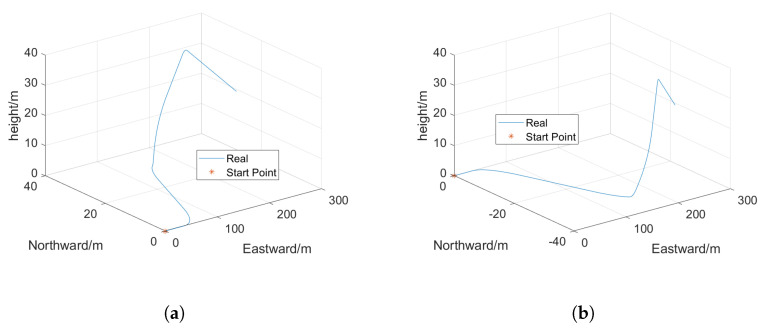
3D simulated pedestrian trajectory. (**a**) 3D simulated trajectories a. (**b**) 3D simulated trajectories b.

In order to verify the rationality and feasibility of the proposed Gait-AUKF pedestrian trajectory localization algorithm, simulation experiments and real data tests were conducted. Real world data is collected using independently developed wearable devices worn by pedestrians. The placement of the equipment is shown in Figure 7. The simulation experiment replicated the real pedestrian movement trajectory, and the visual results are shown in Figure 8.

The simulation system was configured with an SINS data sampling rate of 200 Hz and a GNSS position update rate of 10 Hz. Detailed parameters are provided in Table 3. The initial geographic location was set to 34.0929° north latitude and 108.5374° east longitude, using a north-east-up (NEU) navigation coordinate system with zero initial velocity. Typical measurement errors from both INS and GPS were superimposed on the predefined reference pedestrian trajectory.

The experiment was designed to include comprehensive motion patterns such as uniform linear motion, ascent, descent, acceleration, and turning in order to evaluate the algorithm’s accuracy in estimating pedestrian position and quantify the resulting localization error.

**Figure 9:**
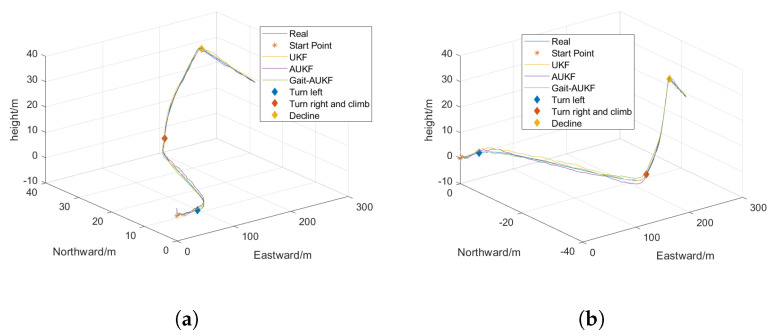
Comparison of 3D simulated trajectories obtained by UKF, AUKF, and Gait-AUKF algorithms. (**a**) 3D simulated trajectories a. (**b**) 3D simulated trajectories b.

**Figure 10:**
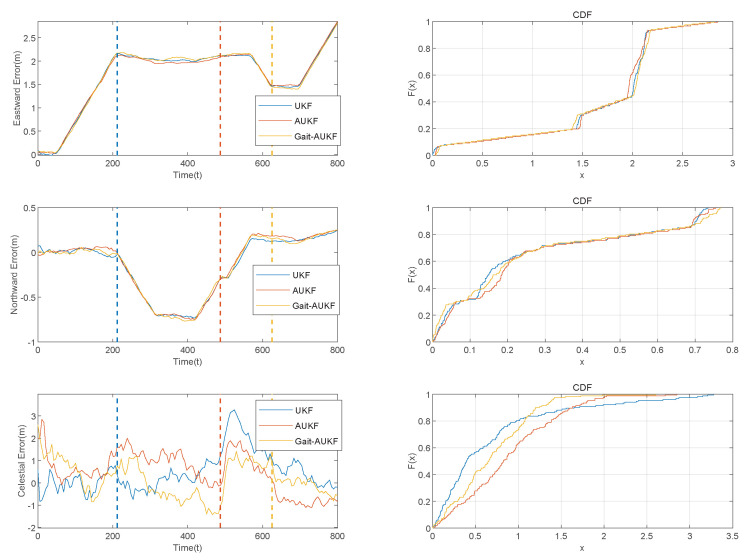
Distance errors and empirical CDF in the East-rth-Up directions for UKF, AUKF, and Gait-AUKF algorithms applied to simulated data.

To validate the robustness of the proposed Gait-AUKF algorithm in complex environments, a simulation experiment was designed with artificially intensified measurement noise to simulate real world scenarios involving sudden noise variations and interference. Under these abnormal noise conditions, a comparative performance test was conducted among three filtering algorithms: UKF, AUKF, and Gait-AUKF, with a focus on analyzing the accuracy of the output navigation trajectories and positional error characteristics. This evaluation aims to assess the adaptability and filtering effectiveness of each algorithm under noisy conditions. Root mean square error (RMSE) and maximum position error (MPE) were adopted as evaluation metrics. The RMSE is defined as:

(40) $$RMSE=\sqrt{\frac{1}{N}\sum_{i=1}^{N}{\left({\hat{X}}_{i}^{k}-X_{i}^{k}\right)}^{2}}$$

where *N* denotes the total number of time points, and ${\hat{X}}_{i}^{k}-X_{i}^{k}$ represents the error between the algorithm output and the ground truth at the *i* time point. The MPE describes the maximum deviation observed under worst case conditions. A comparison of the trajectories estimated by each algorithm is shown in Figure 9, and the distance errors in the east, north, and up directions are presented in Figure 10. The colored diamond markings in Figure 9 represent significant trajectory changes that are about to occur at this time. The blue diamond pattern represents a left turn, the orange diamond pattern represents a climb after a right turn, and the orange diamond pattern represents a descent. The colored dashed lines in Figure 10 also represent the same meaning.

Figure 10 presents a performance comparison of the algorithms in the East-North-Up (ENU) navigation coordinate system. The proposed Gait-AUKF algorithm incorporates gait phase constraints and an adaptive correction mechanism for the measurement noise covariance matrix during iteration, resulting in superior estimation accuracy compared to the conventional UKF and AUKF methods. The results in Table 4 further verify the effectiveness of the proposed algorithm.

**Figure 11:**
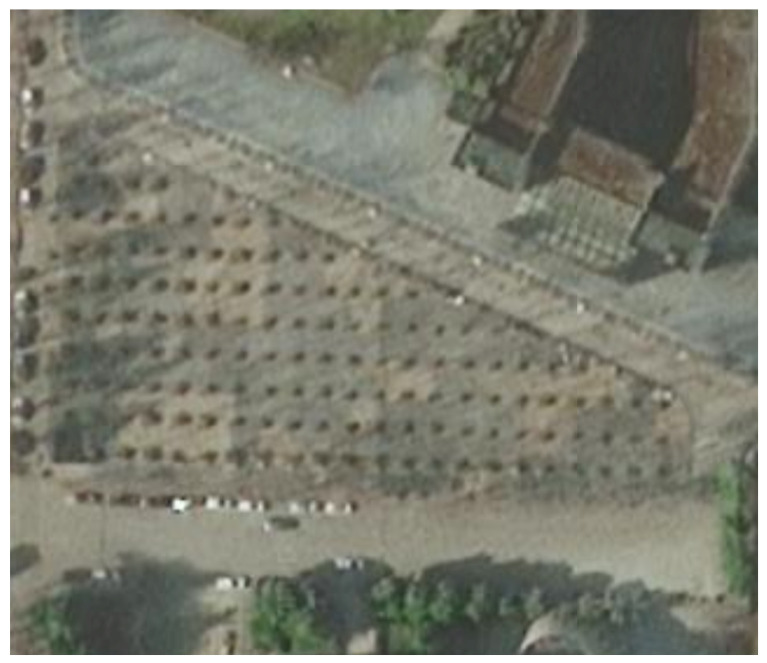
Road scenario for real world data collection.

The real world data collection environment is shown in Figure 11. In this study, a high precision RTK device was used to provide ground truth localization trajectories. The performance of each algorithm was evaluated using RMSE and MPE. Experimental results demonstrate that the Gait-AUKF algorithm maintains better localization accuracy and reliability in real world scenarios.

**Figure 12:**
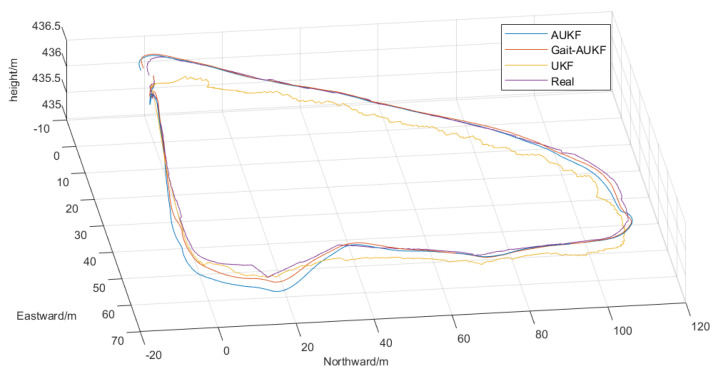
Comparison of 3D real world trajectories obtained by UKF, AUKF, and Gait-AUKF algorithms.

**Figure 13:**
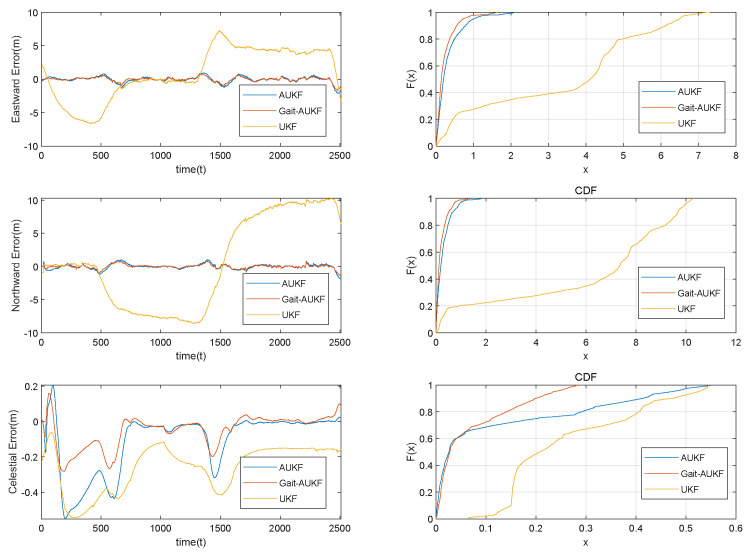
The distance error and empirical CDF of UKF, AUKF, and Gait AUKF algorithms applied to real data in the northeast upward direction.

Figure 12 shows the navigation trajectories obtained from real world measurements, where data collected by the RTK device serve as the ground truth trajectory. The proposed Gait-AUKF algorithm is represented by the orange trajectory, while the blue and yellow lines correspond to the AUKF and UKF algorithms, respectively. As illustrated, the trajectories estimated by the algorithms exhibit certain deviations from the ground truth, which are further quantified in Figure 13. This figure displays the deviations of the three localization algorithms relative to the ground truth in the East-North-Up directions.

In the 3D plane, both the AUKF and Gait-AUKF trajectories align more closely with the ground truth compared to the UKF method, which exhibits noticeable drift over time. Although all three algorithms show deviations in altitude estimation, Gait-AUKF demonstrates significantly smaller errors, indicating superior performance in trajectory localization and tracking. These observations are further supported by the quantitative RMSE and MPE metrics presented in Table 5.

### 4.2. Multi-Source Fusion and Attention-Based Framework for Pedestrian Trajectory Prediction and Planning Decision

This study aims to evaluate the accuracy and practicality of the proposed pedestrian trajectory prediction decision framework. Based on real world road scenarios, we designed a data collection protocol to capture human pose data and location information in real time, thereby constructing a dataset specifically for model training and optimization. This approach enhances the algorithm’s adaptability to diverse and complex environments, using the proposed Gait-AUKF algorithm to filter and fuse pedestrian trajectories collected by wearable devices. The coordinate data generated by GNSS is combined with INS measurement values, and pedestrian gait information is added to form the final input trajectory prediction algorithm dataset. During the training process of SHENet and STMR models, the frame numbers in the models are based on the absolute time obtained from GNSS. Convert the two dimensional coordinates relative to the static image to the absolute position output by Gait-AUKF, and then convert them to plane coordinates based on the relative motion of pedestrians. The ST-MR model outputs multimodal predicted trajectories. When compared with other models, the predicted trajectory with the smallest error from the original trajectory is selected from the multimodal predicted trajectories. Table 6 summarizes the parameter settings used during the training period.

The performance of the trajectory prediction model was evaluated using average displacement error (ADE) and final displacement error (FDE), both inversely related to prediction accuracy, i.e., lower values indicate better performance. ADE measures the average Euclidean distance between the predicted and ground truth trajectories over all time steps:

(41) $$\mathrm{ADE}=\frac{1}{T}\sum_{t=1}^{T}\sqrt{{\left(x_{t}-{\hat{x}}_{t}\right)}^{2}+{\left(y_{t}-{\hat{y}}_{t}\right)}^{2}}$$

Among them, *T* is the total number of time steps, $\left(x_{t},y_{t}\right)$ and $\left({\hat{x}}_{t},{\hat{y}}_{t}\right)$ respectively represent the ground truth and predicted position in the coordinate system established with the predicted point as the origin at time *t*. FDE computes the Euclidean distance specifically at the final predicted time step:

(42) $$\mathrm{FDE}=\sqrt{{\left(x_{t}-{\hat{x}}_{t}\right)}^{2}+{\left(y_{t}-{\hat{y}}_{t}\right)}^{2}}$$

**Figure 14:**
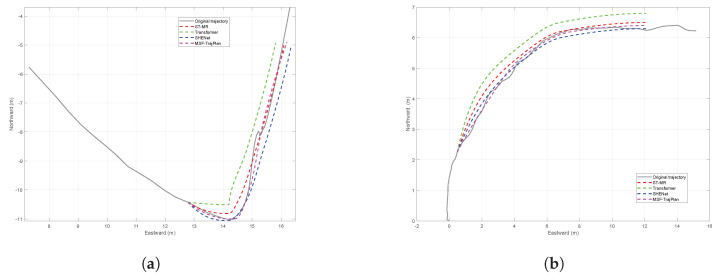
Comparison of actual and predicted pedestrian trajectories. (**a**) Turning path a. (**b**) Turning path b.

During the process of pedestrians walking in a straight line, the range of trajectory changes is not large, and the quality of model prediction cannot be evaluated. Therefore, this article chooses turning intersections with drastic changes to verify the model. As shown in Figure 14, in the nonlinear motion of pedestrians, the predicted trajectories of different methods were compared with the original trajectories. Specifically, the SHENet [22], ST-MR model [21], and proposed method (MSF-TrajPlan) perform more similarly, while the trajectory predicted by the Transformer model shows significant deviation. At the beginning of trajectory prediction, the SHENet model showed good response, but as the time length increased, the ST-MR model became more prominent in long-term trajectories. In contrast, the method proposed in this article performs more stably throughout the entire prediction process. This is because the motion feature encoding mechanism and historical trajectory memory mechanism not only effectively handle the instantaneous changes in pedestrian actions but also dynamically remember trajectory time information, thereby enhancing the smoothness and rationality of the trajectory. Table 7 provide quantitative performance indicators.

**Figure 15:**
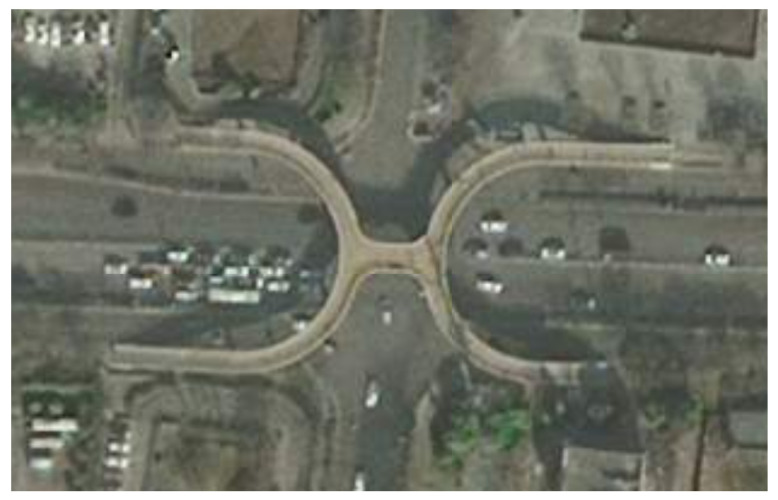
Satellite map of the overpass intersection.

This study models a crosswalk intersection with a pedestrian overpass in an urban area, where start and end points are defined based on satellite imagery to simulate streetcrossing behavior. Since the ground crosswalks are blocked by railings, pedestrians must use the overpass to reach their destinations. A satellite view of the urban intersection is presented in Figure 15.

**Figure 16:**
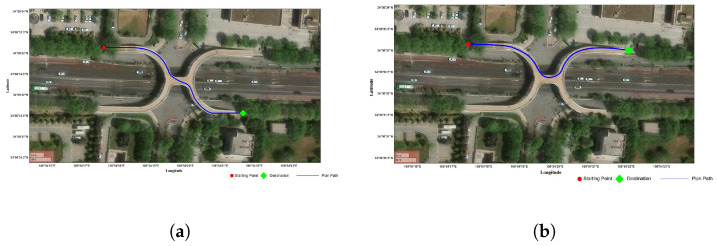
Path planning results for pedestrian overpass crossing. (**a**) Starting point and ending point facing each other. (**b**) Starting point and ending point in the same direction.

In the validation experiment of pedestrian movement through the overpass intersection, the trajectory prediction and planning decision framework produced the results shown in Figure 16. To facilitate interpretation, the trajectories are visualized with red circles indicating the start points, green diamonds denoting the end points, and blue lines representing the predicted paths, allowing clear comparison among trajectories with different endpoints.

**Figure 17:**
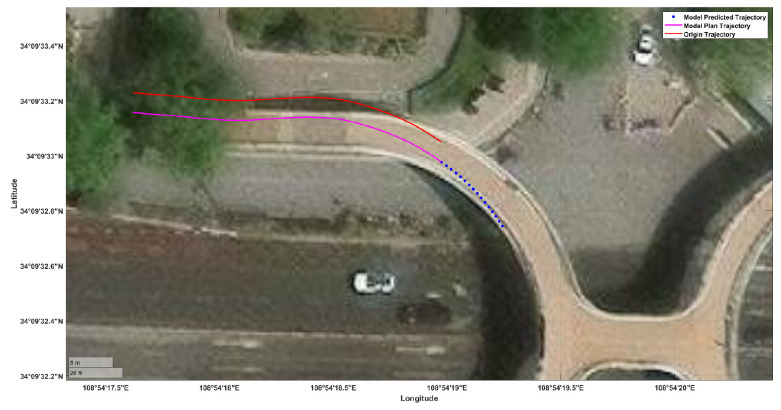
Model based trajectory localization and prediction results in real intersection scenarios.

As shown in Figure 16, the model generates different planned routes based on the destination, with each route starting from the same starting point. Both suggested paths involve crossing pedestrian overpasses and comply with realistic movement restrictions and physical accessibility. Figure 17 shows the difference in trajectory between pure GNSS data without map restrictions and actual roads responsible for urban roads. The GNSS data exhibits certain deviations, while the model constrains the trajectory correctly to the overpass structure and generates stable predicted trajectory points under the guidance of the model.

The ablation experiment results in Table 8 reveal the different effects of the two core modules Gait-AUKF module and A* path planning module on prediction error in this framework. The analysis found that the core function of the Gait-AUKF module is to provide the initial state of the system and the historical trajectory of pedestrians, while the core function of the A* path planning module is to impose physical feasibility and scene structure constraints.

The accuracy of the current state provided by the Gait-AUKF module determines the initial conditions for recursive prediction. Without this module, errors in the decoding process are continuously amplified and accumulated by the recursive model, resulting in severe offset of the predicted endpoint. The function of the A* path planning module is to limit the trajectory provided by A* to physically feasible paths through fusion mechanisms when the decoder generates trajectory segments that may cross obstacles or deviate from feasible areas based on behavioral patterns. This enhances the overall rationality of the trajectory and has a good effect on improving ADE.

## 5. Conclusions

This study proposed a multi-source fusion and attention-based framework for pedestrian trajectory prediction and planning, supported by a novel Gait-AUKF algorithm that adaptively integrates gait phase detection with nonlinear motion modeling. Experimental results demonstrate that the proposed method achieves higher accuracy and robustness compared to UKF, AUKF, LSTM, and Transformer baselines, particularly in turning scenarios and complex urban settings such as overpass intersections. The incorporation of Gait-AUKF and A* path planning significantly improved performance, reducing ADE and FDE by over 68% and 71%, respectively. The framework provides a reliable solution for trajectory prediction in dynamic pedestrian environments.

## Figures and Tables

**Table 1:** JY901S parameters.

Unit	Dimensions (Axis)	Dynamic Range	Sensitivity	Word Length
Accelerometer	3	±16 g	0.0005 (g/LSB)	16 bits
Gyroscope	3	±2000°/s	0.061 (°/s)/(LSB)	16 bits
Magnetometer	3	±2 Gaituss	6.67 nT/LSB	16 bits

**Table 2:** E108-GN04D parameters.

Unit	Cold Start	Hot Start
localization time	28 s	1 s
Sensitivity	−148 dBm	−159 dBm
Accuracy	2 m-CEP, 0.05 m/s-RMS	1.5 m-CEP, 0.05 m/s-RMS

**Table 3:** Simulation sensor parameter settings.

Simulation Parameters (Unit)	Parameter Settings
SINS Gyroscope Bias (°/s)	0.05
SINS Gyroscope Random Walk (°/$\sqrt{h}$ )	0.65
SINS Accelerometer Bias (mg)	40
SINS Accelerometer Random Walk (ug/$\sqrt{\mathrm{Hz}}$ )	600
SINS Sampling Rate (Hz)	200
GPS Ranging Error (m)	2.0
GPS Velocity Error (m/s)	0.1
GPS Sampling Rate (Hz)	10

**Table 4:** Evaluation metrics of different algorithms for simulated data.

Method	Direction	RMSE (m)	MPE (m)
UKF	East	2.3734	2.8428
	North	2.6592	2.9859
	Up	3.2435	7.4907
AUKF	East	2.3259	2.8134
	North	2.4349	2.6039
	Up	3.7291	6.8660
Gait-AUKF	East	2.2368	2.4380
	North	2.0628	2.2753
	Up	3.1792	6.3275

**Table 5:** Evaluation metrics of different algorithms for real world data.

Method	Direction	RMSE (m)	MPE (m)
UKF	East	2.0396	2.1107
	North	1.1675	1.8332
	Up	6.9158	0.7143
AUKF	East	0.4796	3.1584
	North	0.4148	1.6458
	Up	0.2288	0.7295
Gait-AUKF	East	0.3551	1.7461
	North	0.3058	1.0275
	Up	0.1165	0.3938

**Table 6:** Training model parameters.

Parameter	Value
Epochs	1000
Batch Size	16
Initial LR (Feature)	$10^{-3}$
Initial LR (Memory)	$10^{-4}$
Initial LR (Trajectory)	$10^{-3}$
Final LR (All Modules)	$10^{-6}$
Optimizer	ADAM

**Table 7:** Performance of trajectory prediction of various models in actual road scenarios.

Model	ADE	FDE
ST-MR	0.97	1.20
Transformer	1.16	1.39
SHENet	1.01	1.24
MSF-TrajPlan	0.93	1.16

**Table 8:** Ablation study in the overpass intersection scenario (unit: meters).

Model	ADE	FDE
Without A* Planning	1.61	2.49
Without Gait-AUKF	1.36	3.74
Without Gait-AUKF and A*	2.92	5.19
With Gait-AUKF and A*	0.93	1.16

## Data Availability

The data supporting the reported results in this study are not publicly available due to privacy restrictions.
